# Safeguards of Neurotransmission: Endocytic Adaptors as Regulators of Synaptic Vesicle Composition and Function

**DOI:** 10.3389/fncel.2017.00320

**Published:** 2017-10-11

**Authors:** Natalie Kaempf, Tanja Maritzen

**Affiliations:** Molecular Physiology and Cell Biology Section, Leibniz-Forschungsinstitut für Molekulare Pharmakologie (FMP), Berlin, Germany

**Keywords:** AP180, stonin2, sorting, recycling, release site clearance, presynapse

## Abstract

Communication between neurons relies on neurotransmitters which are released from synaptic vesicles (SVs) upon Ca^2+^ stimuli. To efficiently load neurotransmitters, sense the rise in intracellular Ca^2+^ and fuse with the presynaptic membrane, SVs need to be equipped with a stringently controlled set of transmembrane proteins. In fact, changes in SV protein composition quickly compromise neurotransmission and most prominently give rise to epileptic seizures. During exocytosis SVs fully collapse into the presynaptic membrane and consequently have to be replenished to sustain neurotransmission. Therefore, surface-stranded SV proteins have to be efficiently retrieved post-fusion to be used for the generation of a new set of fully functional SVs, a process in which dedicated endocytic sorting adaptors play a crucial role. The question of how the precise reformation of SVs is achieved is intimately linked to how SV membranes are retrieved. For a long time both processes were believed to be two sides of the same coin since Clathrin-mediated endocytosis (CME), the proposed predominant SV recycling mode, will jointly retrieve SV membranes and proteins. However, with the recent proposal of Clathrin-independent SV recycling pathways SV membrane retrieval and SV reformation turn into separable events. This review highlights the progress made in unraveling the molecular mechanisms mediating the high-fidelity retrieval of SV proteins and discusses how the gathered knowledge about SV protein recycling fits in with the new notions of SV membrane endocytosis.

## Introduction

Communication between neurons is based on the release of neurotransmitters at the presynapse which bind to postsynaptic receptors thereby inducing electrical signals. These neurotransmitters are stored within synaptic vesicles (SVs) which dock at specialized release sites in close proximity to Ca^2+^ channels within the presynaptic active zone. Incoming action potentials lead to an influx of Ca^2+^ into the presynapse and thereby trigger the fusion of docked SVs and thus neurotransmitter release. To allow for sustained neurotransmission SV exocytosis needs to be tightly balanced by endocytosis for three main reasons: (1) the expansion of the active zone needs to be reversed to preserve the alignment of the release sites with postsynaptic receptors and to restore membrane tension; (2) the release sites have to be cleared from exocytosed material to be available for new rounds of SV fusion; and (3) since synapses are mostly located far away from the cell body, SV membrane proteins have to be retrieved for the local reformation of SVs to replenish the SV pool.

## SV Membrane Protein Composition

To recycle SVs is in fact a daunting task since SVs need to be reliably equipped with a complex set of proteins at defined stoichiometry to efficiently maintain neurotransmission. Pioneering biochemical studies on SV composition by Takamori et al. (2006) indicated the presence of about 80 different integral membrane proteins on SVs, at least 40 of them known SV residents. Most important for neurotransmitter release are those SV proteins mediating the uptake of neurotransmitters i.e., the different neurotransmitter transporters such as VGLUT for glutamate uptake, VGAT for GABA uptake and VMAT for monoamine uptake, as well as the vacuolar H^+^-ATPase, the Ca^2+^ sensor Synaptotagmin1 and the fusion protein Synaptobrevin2 (also known as VAMP2). In addition, the transmembrane proteins SV2 and Synaptophysin1 are needed for efficient neurotransmission. This is partly due to their contribution to the sorting of Synaptotagmin1 respectively Synaptobrevin2 as we will discuss in more detail in the sections “Sorting of Synaptobrevin2” and “Sorting of Synaptotagmin1”.

Some of these proteins are found in high copy numbers on SVs like Synaptobrevin2 and Synaptophysin1, whereas others such as Synaptotagmin1 and SV2 are present at lower amounts (Takamori et al., 2006; Mutch et al., 2011). This was mainly established by two studies employing very different techniques. Takamori et al. (2006) relied on quantitative western blotting to determine protein levels in SV preparations using recombinant protein standards for calibration ; Mutch et al. (2011) used TIRF microscopy on purified SVs which were labeled with fluorescent antibodies to determine the protein amounts on individual vesicles. While both studies are in overall agreement, the exact copy numbers they report often vary. The highest discrepancy was observed for the abundant SV protein Synaptobrevin2 whose reported copy number ranges from 11 to 70 (Takamori et al., 2006; Mutch et al., 2011). As the authors point out, the microscopical study might be biased to underestimate SV protein copy numbers since in intact SVs not all proteins might be equally accessible to antibody binding e.g., due to blocking interactions. However, both studies agree that there are only 1–2 copies per SV of the vacuolar H^+^-ATPase (Takamori et al., 2006; Mutch et al., 2011) posing the question how the necessary extremely accurate sorting is achieved. While biochemical experiments do not allow to determine intervesicle variability, the micoscopy-based single molecule quantification approach showed significant intervesicle variability in the high copy number SV proteins Synaptobrevin2 and Synaptophysin1, while Synaptotagmin1, VGLUT1, SV2 and the vacuolar H^+^-ATPase displayed much less variation (Mutch et al., 2011) again arguing for highly precise sorting mechanisms.

## Principle Mechanisms for SV Protein Sorting

**Figure 1:**
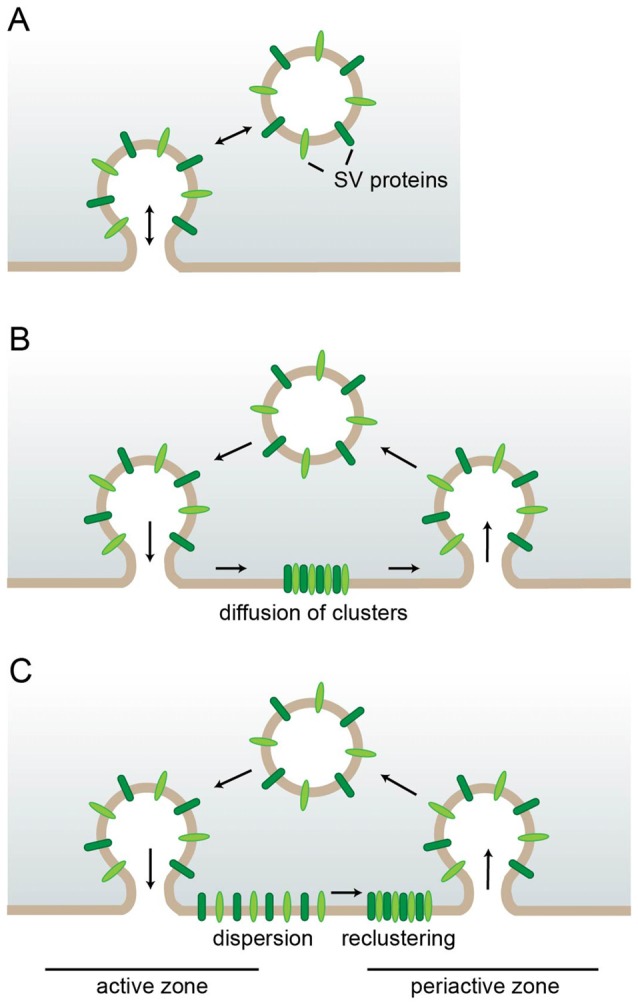
Requirements for synaptic vesicle (SV) protein sorting in different endocytic modes. **(A)** In kiss-and-run endocytosis the SV only transiently fuses. Closure of the fusion pore restores the SV with unaltered protein composition eliminating the need for sorting. **(B,C)** After full collapse fusion SV proteins strand in the presynaptic membrane. There they can either stay associated and diffuse as cluster out of the active zone reducing the need for individual sorting adaptors **(B)** or they can first disperse and later recluster with the aid of self-aggregation mechanisms and specific sorting adaptors **(C)**.

So how is SV protein sorting accomplished with the high-fidelity necessary to preserve the composition and stoichiometry of SVs during SV recycling? The extent to which protein sorting is necessary to achieve this goal depends greatly on the mode of endocytosis employed (Figure 1). During “kiss and run” endocytosis for instance the fusing vesicle releases its content via a transient fusion pore which rapidly closes again (Ceccarelli et al., 1972) allowing the vesicle to depart with its composition still intact obliterating the need for protein sorting (Figure 1A). However, while there is strong support for “kiss and run” to operate in secretory cells such as chromaffin cells (Albillos et al., 1997; Alés et al., 1999; Chiang et al., 2014), its role in central nervous system (CNS) synapses is still intensely debated. While a number of studies mainly argue for “kiss and run” based on the rapid endocytosis kinetics the authors observe (Sun et al., 2002; Gandhi and Stevens, 2003; Wu et al., 2005; He et al., 2006), the most convincing arguments come from high-resolution imaging of SVs labeled with the fluorescent membrane dye FM1-43. The only partial loss of fluorescence which was frequently observed after SV fusion is most consistent with a small fusion pore which rapidly closes again (Aravanis et al., 2003; Richards et al., 2005; Harata et al., 2006). However, most studies agree that the speed of endocytosis varies, e.g., depending on the stimulation paradigm (Sun et al., 2002; Gandhi and Stevens, 2003; Richards et al., 2005; Harata et al., 2006; Kononenko et al., 2014; Delvendahl et al., 2016; Soykan et al., 2017); and in many cases neurotransmitter release rather seems to entail the full collapse of SVs into the presynaptic membrane as first observed at the neuromuscular junction (Heuser and Reese, 1973; Heuser, 1989; Koenig and Ikeda, 1996) and later also demonstrated for CNS nerve terminals (Sankaranarayanan and Ryan, 2000, 2001; Li and Murthy, 2001; Klyachko and Jackson, 2002; Zenisek et al., 2002; Aravanis et al., 2003; Gandhi and Stevens, 2003; Richards et al., 2005).

In this scenario the extent to which the surface-stranded SV proteins remain associated determines how much sorting is necessary. If the entire set of SV proteins stayed together as a package for retrieval, only a single sorting adaptor would be needed (Figure 1B). However, this model is at odds with several observations: first, the loss of specific endocytic sorting adaptors selectively impairs the retrieval of individual SV proteins (Kononenko et al., 2013; Koo et al., 2015). Second, advanced microscopy approaches have shown surface-stranded SV proteins to be mobile within the presynaptic membrane and to intermix with newly exocytosed SV proteins (Fernandez-Alfonso et al., 2006). In fact, freshly exocytosed and subsequently endocytosed SV proteins are not identical (Wienisch and Klingauf, 2006). These findings are consistent with a recent study demonstrating an initial rapid diffusional dispersion of newly exocytosed SV proteins out of the active zone followed by confinement to prevent loss into the axon and by slow reclustering at the periactive zone (Gimber et al., 2015; Figure 1C). The advantage of this sequence of events is the rapid clearance of release sites from exocytosed material which presumably works more efficiently for solitary proteins than for a large protein cluster which needs to find its way through the dense cytomatrix of the active zone. However, it also necessitates more elaborate mechanisms for confining, sorting and reclustering the dispersed SV proteins which could either involve a plethora of distinct sorting adaptors for the individual SV proteins or a combination of a smaller number of adaptors working on SV proteins which self-assemble into mixed clusters. In fact, interactions between the SV proteins Synaptotagmin1 and SV2 (Kaempf et al., 2015), and Synaptobrevin2 and Synaptophysin1 (Gordon et al., 2011) have been suggested to play important roles during sorting as we will discuss in more detail later.

In addition, the two SV proteins VGLUT1 (Pan et al., 2015) and Synaptotagmin1 (Koch and Holt, 2012) have been proposed to serve as “hubs” that orchestrate the retrieval of additional SV proteins. When VGLUT endocytosis was perturbed, also the uptake of other SV proteins such as SV2 and synaptophysin was slowed down (Pan et al., 2015). The fact that Synaptotagmin1 retrieval was not altered upon VGLUT1 depletion implies that both proteins might function in parallel. In flies also AP180 has been proposed to cluster different SV proteins for recycling (Vanlandingham et al., 2014), while in mammals its role appears to be more restricted to Synaptobrevin2 sorting (Koo et al., 2015) as discussed in the section “Sorting of Synaptobrevin2”.

## Modes of SV Endocytosis and Reformation

**Figure 2:**
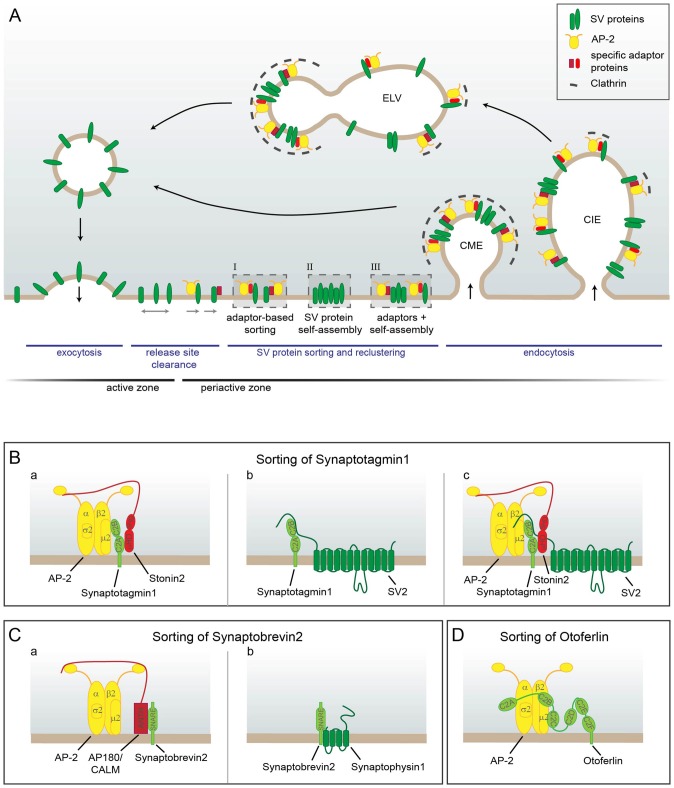
Model of SV protein reclustering and sorting at the presynapse. **(A)** After full collapse fusion freely diffusing SV proteins are confined and recaptured by endocytic sorting adaptors at the periactive zone to allow for release site clearance. At the plasma membrane SV proteins might either be clustered by AP-2 and additional cargo-specific adaptor proteins (I), they might interact with each other and thereby self-assemble into clusters (II) or form mixed clusters of self-assembled SV proteins together with sorting adaptors (III). These clusters can directly be endocytosed from the plasma membrane by Clathrin-mediated endocytosis (CME) to reform SVs. However, cargo-specific sorting proteins together with AP-2 and Clathrin can also operate on endosomal-like vacuoles (ELVs) after Clathrin-independent endocytosis (CIE) to recycle SVs with correct protein composition. **(B–D)** Sorting of individual SV proteins. **(B)** The precise sorting of Synaptotagmin1 can be accomplished by three possible mechanisms. **(a)** Synaptotagmin1 associates with AP-2μ2 via its C2B domain. However, this interaction does not suffice for efficient Synaptagmin1 retrieval. The specific adaptor protein Stonin2 is needed to strengthen the link between AP-2 and Synaptotagmin1 by interacting simultaneously with Synaptotagmin1’s C2A domain and the AP-2α ear. **(b)** Synaptotagmin1 can also be sorted by association with SV2, another SV protein. The N-terminus of SV2 binds to the C2B domain of Synaptotagmin1 and thereby facilitates correct Synaptotagmin1 sorting. **(c)** As Stonin2 and SV2 interact with distinct C2 domains of Synaptotagmin1, both proteins might bind at the same time to the Ca^2+^ sensor and link it to AP-2 to enable its precise retrieval. **(C)** Free Synaptobrevin2 can also be sorted by multiple mechanisms. **(a)** Synaptobrevin2 is recognized by the specific sorting adaptors AP180 and CALM via their ANTH domain that interacts with the SNARE domain of Synaptobrevin2 and with the plasma membrane. Via the unstructured C-terminus AP180 and CALM associate with AP-2 and Clathrin thereby linking Synaptobrevin2 to the CME machinery. **(b)** Synaptobrevin2 can also be sorted by association with the SV protein Synaptophysin1 via their transmembrane domains. **(D)** In inner hair cells Otoferlin is directly sorted by AP-2 via the interaction of di-leucine motifs within the linker regions between the C2A-C2B-C2C domains of Otoferlin with AP-2α/σ2. In addition, Otoferlin associates with AP-2μ2. However, the exact interaction sites are not clear yet.

But how are SVs retrieved after full collapse fusion? For a long time most data seemed to point to Clathrin-mediated endocytosis (CME) from the plasma membrane as the main mechanism for SV retrieval (Heuser and Reese, 1973; Granseth et al., 2006; Dittman and Ryan, 2009; Saheki and De Camilli, 2012). In this type of endocytosis adaptor proteins like AP-2, which bind to the phosphoinositide PI(4,5)P_2_ and to cargo molecules, recruit Clathrin triskelia to form a Clathrin coat on the membrane. Accessory endocytic factors including Epsin and BAR-domain-containing proteins like Endophilin, which have membrane bending and stabilizing activity, act in conjunction with the Clathrin coat to progressively invaginate the plasma membrane. The resulting vesicle is finally pinched off by the action of the large GTPase and mechano-enzyme Dynamin. Eventually, by uncoating, reacidification and refilling with neurotransmitters the endocytosed Clathrin-coated vesicle turns into a new fully functional SV thereby completing the cycle of SV recycling (Dittman and Ryan, 2009). In this process adaptor-mediated cargo sorting, membrane retrieval and SV reformation are inextricably linked (Figure 2A).

However, since CME operates on the time scale of tens of seconds, this tight link might actually be problematic. While SV reformation can easily occur with some delay as synapses contain a sufficiently large SV reservoir, the restoration of the release sites by immediate membrane retrieval might well become rate limiting (Neher, 2010). Therefore, a two-step mechanism would be much better suited to allow neurons greater adaptability of their SV release thereby enabling them to cope with a wider range of stimuli. In line with this reasoning highly time-resolved electron microscopy of optogenetically stimulated and high-pressure frozen neurons uncovered the so-called ultrafast endocytosis (UFE) mode. Within 50–100 ms post-fusion endosomal-like vacuoles (ELVs), which correspond in size to multiple SVs, form next to the active zone in an Actin- and Dynamin-dependent, but Clathrin-independent manner rapidly restoring the membrane area (Watanabe et al., 2013). Subsequently, these ELVs are consumed by Clathrin-dependent SV reformation from the ELV membrane (Watanabe et al., 2014). Thus, membrane retrieval and SV reformation are here two separable events (Figure 2A).

Later studies confirmed that synaptic membrane retrieval can operate in absence of Clathrin and AP-2 (Kononenko et al., 2014; Soykan et al., 2017), while the Clathrin machinery is indeed needed to regenerate SVs from ELVs (Kononenko et al., 2014; Jung et al., 2015; Koo et al., 2015). Depletion of Clathrin, AP-2 or the endocytic adaptor AP180 leads to an accumulation of ELVs since they are not efficiently consumed in absence of CME (Kononenko et al., 2014; Jung et al., 2015; Koo et al., 2015; Soykan et al., 2017). However, synaptic Clathrin-independent endocytosis (CIE) was found to operate over a broader time range than UFE. In fact, upon stimulation with more than two action potentials, the majority of SV membrane seems not to be internalized with subsecond kinetics, but rather over several seconds (Soykan et al., 2017). These results suggest that the retrieval capacity of UFE might be limited to the amount of SV membrane exocytosed in response to stimulation with single or very few action potentials. Additionally exocytosed membrane area appears then to be endocytosed via a less rapid Clathrin-independent endocytic mode (Soykan et al., 2017).

The finding that presynaptic AP-2-dependent Clathrin-mediated vesicle formation operates at ELVs came as a surprise since AP-2 had previously been exclusively described as plasma membrane-based endocytic adaptor, while related complexes like AP-1 are known to facilitate Clathrin coat assembly on endosomal vesicles (Robinson, 2015). Since AP-2 as well as a number of other CME proteins rely on the mainly plasma membrane-localized phosphoinositide PI(4,5)P_2_ for their recruitment (Posor et al., 2015), it will be interesting to dissect the lipid composition of ELVs as opposed to other endosomal compartments.

However, since many studies have found SV proteins to accumulate at the plasma membrane upon perturbation of their sorting (Gordon et al., 2011; Kononenko et al., 2013; Koo et al., 2015), it is hardly conceivable that the CME machinery operates exclusively on ELVs in the presynapse. In fact, while Clathrin and AP-2 are in principle dispensable for SV endocytosis, they were shown to contribute to membrane retrieval after sustained low-frequency stimulation (Kononenko et al., 2014). Besides, proteins of the CME machinery appear to have additional functions at the plasma membrane by facilitating release site clearance prior to endocytosis (Sakaba et al., 2013; Gimber et al., 2015; Jung et al., 2015) as discussed in more detail in the section “Additional Functions for CME-Associated Adaptors: Release Site Clearance”. Therefore, the following model has been proposed (Soykan et al., 2017): CME proteins facilitate the movement of SV proteins out of the active zone and concentrate them at the periactive zone for endocytosis. How rapidly endocytosis proceeds likely depends on neuronal activity. The speed of endocytic fission could e.g., be influenced by changes in Ca^2+^ concentration and Dynamin1 phosphorylation. Upon slow fission there is likely sufficient time for Clathrin coats to assemble on the plasma membrane, while upon fast fission Clathrin might only later be recruited to ELVs which presumably retain a plasma-membrane-like lipid composition (Figure 2A).

## Endosomal Sorting of SV Proteins

The fact that SV proteins are at least partially sorted at the level of endosomal compartments has in fact long been known. Takamori et al. already noted that SVs carry fusion proteins not only for exocytosis, but also for fusion with endosomes (Takamori et al., 2006) suggesting that they contact endosomal compartments at some point. In line with this, Hoopmann et al. (2010) reported endosomal sorting to be involved in the recycling of the readily releasable SV pool, and also others have shown that recycling SVs can pass through endosomes (Uytterhoeven et al., 2011; Korber et al., 2012). In fact, loss of the endosomally localized adaptor complexes AP-1 and AP-3 impairs SV recycling and leads to neurological alterations such as reduced motor coordination and impaired long-term spatial memory (Blumstein et al., 2001; Glyvuk et al., 2010). Both complexes were shown to assist in the reformation of SVs from activity-dependent bulk endosomes which have been observed at central synapses after very high intensity stimulation (Cheung and Cousin, 2012). Biogenesis of lysosome-related organelles complex 1 (Bloc-1), an endosomal sorting complex involved in transport from the early endosome to lysosomes and lysosome-related organelles, was likewise shown to be involved in cargo sorting to SVs (Newell-Litwa et al., 2009; Di Giovanni and Sheng, 2015). The topic of endosomal sorting is closely linked to the question of SV protein quality control as it offers a possibility to shuttle SV proteins into degradative pathways, a field which is actively researched at the moment. For the future it will be important to unravel the precise connection between the ELVs arising by UFE/CIE and the different types of endosomal/lysosomal/autophagosomal compartments to understand in detail which path SV membranes and proteins follow at which point of their live to facilitate the regeneration of fully functional SVs.

## CME-Associated Adaptors in SV Protein Sorting

Cargo adaptors are a universal means to sort transmembrane proteins into budding vesicles (Traub and Bonifacino, 2013). As mentioned earlier, in the context of CME cargo selection is inextricably linked to the process of membrane retrieval since the main endocytic adaptor AP-2 does not only bind to the tails of numerous transmembrane cargo proteins but also serves as linker to the Clathrin coat and to a plethora of accessory endocytic factors involved in membrane remodeling (Wieffer et al., 2009; McMahon and Boucrot, 2011). AP-2 and also the additional endocytic adaptors which are dedicated to the uptake of very specific cargo proteins have the same overall structure (Traub and Bonifacino, 2013), a combination of folded domains and unstructured regions. Via the folded domains they bind to their cargo proteins and often also to phosphoinositides to facilitate their recruitment to sites of endocytosis. The unstructured regions generally contain diverse motifs for binding to other endocytic factors. In this way the adaptor proteins firmly link their bound cargo to the endocytic machinery and facilitate its internalization.

## Sorting of Multiple SV Proteins by AP-2

AP-2 is the main cargo-adaptor in CME by binding to a diverse set of cargo proteins as well as orchestrating the assembly of the Clathrin coat (Wieffer et al., 2009; McMahon and Boucrot, 2011). AP-2 is a heterotetrameric complex composed of the large α and β2 subunits (100 kDa), the medium-sized μ2 subunit (50 kDa) and the small σ2 subunit (17 kDa) which binds cargo in three ways: Via its μ subunit it binds tyrosine-based motifs of the type YXXφ, where X indicates any amino acid and φ an amino acid with a bulky hydrophobic side chain. The α and σ2 subunits provide the binding site for acidic cluster di-leucine-based motifs of the type [DE]XXXL[LI] (brackets indicate that either amino acid is allowed at this position). Finally, the μ2 subunit can also bind to C2 domains (Traub and Bonifacino, 2013).

When looking into the sequences of SV proteins, it was initially surprising that some very important ones such as Synaptobrevin2 do not have any of the motifs recognized by AP-2. They were later found to require the mentioned additional specific endocytic adaptors. But a number of SV proteins contain classical AP-2 recognition motifs. VGLUT1 for instance was shown to have di-leucine based motifs (Foss et al., 2013), SV2 binds to AP-2 via a tyrosine-based motif (Yao et al., 2010) and Synaptotagmin1 associates with AP-2 via its C2B domain (Haucke et al., 2000). However, all these SV proteins in contrast to most other AP-2 cargo proteins do not rely solely on AP-2 for their connection to the endocytic machinery, but interact with additional endocytic factors. VGLUT1 was shown to bind to Endophilin (Voglmaier et al., 2006), SV2 interacts with Eps15 and Amphiphysin (Yao et al., 2010), and Synaptotagmin1 binds to the endocytic adaptor Stonin2 (Jung et al., 2007).

In inner hair cells recently a new SV cargo of AP-2 was identified, the C2 domain containing protein Otoferlin, which is crucial for SV replenishment at the ribbon synapse (Pangršič et al., 2010, 2012). Otoferlin associates via di-leucine motifs with AP-2 α/σ2 hemicomplexes and also binds μ2 (Figure 2D). In absence of AP-2 otoferlin levels were drastically reduced, and the remaining protein was stranded at the plasma membrane (Jung et al., 2015). Consistently, inner hair cell specific AP-2 knockout (KO) mice suffered from hearing impairment (Jung et al., 2015).

Constitutive AP-2 KO mice die already during embryonal development (Mitsunari et al., 2005), while brain-specific AP-2 loss is lethal within the first weeks after birth (Kononenko et al., 2017). The fact that brain-specific AP-2 KO mice are viable for about 3 weeks might be consistent with the reported NCAM-dependent switch from AP-3 to AP-2 during synapse maturation (Shetty et al., 2013). As described in the paragraph on endocytic modes, under most stimulation conditions loss of AP-2 does not impair SV protein retrieval from the plasma membrane (Kononenko et al., 2014; Jung et al., 2015; Soykan et al., 2017), however it does cause the accumulation of large ELVs and a reduction in SVs (Kononenko et al., 2014; Jung et al., 2015), presumably because the ELVs cannot be efficiently converted into SVs.

## Sorting of Synaptobrevin2

Synaptobrevin2 is crucial for the fusion of SVs (Schoch et al., 2001; Deák et al., 2004). As a vesicular soluble NSF attachment protein receptor (v-SNARE) protein it forms via its SNARE domain a tight four-helical bundle together with the two plasma-membrane localized target SNARE (t-SNARE) proteins SNAP25 and Syntaxin1. Complex formation between the SNARE proteins is essential to bring SV and plasma membrane into close proximity and thereby drive their fusion (Jahn and Fasshauer, 2012). Loss of Synaptobrevin2 nearly abolishes Ca^2+^-induced release and therefore causes immediate postnatal lethality (Schoch et al., 2001). In addition to its SNARE domain Synaptobrevin2 contains a carboxy-terminal transmembrane domain for membrane anchoring and a short proline-rich amino-terminal domain, all of them devoid of motifs for AP-2 binding.

Ideas for alternative proteins to facilitate Synaptobrevin2 internalization came among others (for more details see our previous review; Maritzen et al., 2012) from studies in *C. elegans*. Synaptobrevin displayed a diffuse localization and impaired endocytosis in nematodes mutant for the protein Unc11 (Nonet et al., 1999; Dittman and Kaplan, 2006) which has two homologs in mammals, called assembly protein 180 (AP180; gene name: SNAP91) and Clathrin assembly lymphoid myeloid leukemia (CALM; gene name: PICALM). These proteins conform to the outlined structure of adaptor proteins by having a folded module, the highly conserved AP180 N-terminal homology (ANTH) domain, and an unstructured sequence of 150–600 amino acids harboring a number of AP-2- and Clathrin-binding motifs (Lindner and Ungewickell, 1992; Morris et al., 1993; Hao et al., 1999; Maritzen et al., 2012; Moshkanbaryans et al., 2016). The ANTH domain represents an α-helical structure containing a lipid binding site which mediates the association of AP180 and CALM with PI(4,5)P_2_ and therefore their membrane recruitment (Ford et al., 2001). AP180 was originally discovered as a prominent component of Clathrin-coated vesicles which were purified from brain (Ahle and Ungewickell, 1986; Keen and Black, 1986; Kohtz and Puszkin, 1988). In fact, it was soon recognized that AP180 is an exclusively brain-expressed protein (Puszkin et al., 1992; Zhou et al., 1992) which localizes specifically to the presynapse (Yao et al., 2002, 2005; Koo et al., 2015) in line with a potential sorting function for Synaptobrevin2. CALM on the other hand is ubiquitously expressed (Dreyling et al., 1996) and was also found at the postsynapse (Yao et al., 2005). It is best known nowadays for being linked to Alzheimer’s disease (Harold et al., 2009), even though the contribution of neuronally expressed CALM to the disease is not fully understood yet. While CALM and AP180 display 80% amino acid identity within their ANTH domain, they differ substantially in the length of their unstructured region and the specific motifs they contain for interacting with endocytic proteins (Maritzen et al., 2012).

First evidence that AP180 and CALM play a role in Synaptobrevin2 sorting at mammalian synapses came from experiments with cultured hippocampal neurons which had been depleted for AP180 and/or CALM by siRNA and were probed with pHluorin-based reporters (Koo et al., 2011). pHluorin is a pH-sensitive variant of green fluorescent protein (GFP) which can visualize endocytosis kinetics and levels of surface-stranded SV proteins when it is fused to the lumenal tail of the SV protein in question (Miesenbock et al., 1998; Miesenbock, 2012; Kavalali and Jorgensen, 2014). Its fluorescence will be quenched while the fusion protein resides within acidic SVs, but lights up upon exposure to neutral medium following exocytosis. Subsequent endocytosis and reacidification quench the fluorescence again. The decaying fluorescence signal is used as a surrogate measure for endocytosis, even though, strictly speaking, it is vesicular reacidification which is monitored. This is a critical factor when assessing endocytic kinetics with pHluorin sensors since they cannot detect anything proceeding faster than vesicular reacidification (which requires approximately 3–4 s (Atluri and Ryan, 2006)) and thereby in the past led to an underestimation of the speed of endocytosis (Soykan et al., 2017). However, newer studies have circumvented this problem by rendering the pHluorin-based detection of endocytosis independent of pH changes within the endocytosed vesicle by a combination of quenching of newly exocytosed pHluorin-fused SV proteins and post-endocytic blocking of vesicular reacidification (Soykan et al., 2017). Another caveat when employing pHluorin sensors lies in the necessity for transfection and the possible artifacts caused by overexpression, however use of the neuron-specific synapsin promotor normally achieves sufficiently low expression levels (for an overview of the strengths and weaknesses of the methodologies commonly used to study SV recycling please see Kononenko and Haucke, 2015).

Imaging of Synaptobrevin2-pHluorin indicated its selective accumulation at the presynaptic surface while VGLUT1-pHluorin did not differ in the depleted neurons (Koo et al., 2011). Combined depletion of AP180 and CALM aggravated the surface retention suggesting that they have partially redundant functions in Synaptobrevin2 sorting (Koo et al., 2011).

However, the great abundance of Synaptobrevin2 on SVs posed the question whether for this SV protein high-fidelity sorting is actually crucial *in vivo*, especially since one to three SNARE complexes had been postulated to be sufficient for SV fusion (Mohrmann et al., 2010; van den Bogaart et al., 2010; Sinha et al., 2011). To address this question, Koo et al. (2015) generated AP180 KO mice. Consistent with previous data, neurons from these mice exhibited a surface accumulation of Synaptobrevin2 that was aggravated by depletion of CALM. CALM was in fact upregulated in the AP180 KO mice further underlining the partial redundancy between AP180 and CALM. However, even in the face of CALM upregulation, AP180 KO mice showed strong impairments arguing that CALM cannot fully compensate for loss of AP180. AP180 KO mice lagged behind in growth and died either around the time of weaning or during the following 4 months. During their life time they showed behavioral abnormalities and spontaneous epileptic seizures (Koo et al., 2015). These seizures are due to an imbalance between excitatory and inhibitory neurotransmission arising from a more pronounced impairment of inhibitory transmission. Inhibitory neurons are known to be more tonically active and therefore likely require more rounds of SV recycling. Thus, sorting defects should have stronger consequences in the more active inhibitory neurons. In fact, phluorin experiments demonstrated a larger Synaptobrevin2 surface accumulation in VGAT-positive neurons, while silencing neuronal activity rescued the surface retention (Koo et al., 2015). Consistent with the surface accumulation of Synaptobrevin2, less v-SNARE could be detected on immunoisolated inhibitory SVs likely accounting for the decreased inhibitory neurotransmission (Koo et al., 2015). Thus, high-fidelity Synaptobrevin2 sorting is in fact needed *in vivo* to maintain efficient neurotransmission. The about 70 copies of Synaptobrevin2 on SVs constitute obviously not just a huge safety margin, but more copies of Synaptobrevin2 than previously appreciated seem to be necessary to mediate efficient SV fusion and therefore need to be meticulously retrieved post-fusion.

Biochemical and structural studies unraveled the molecular basis for AP180/CALM-based sorting of Synaptobrevin2 (Koo et al., 2011; Miller et al., 2011). Surprisingly, no peptide motif is involved in contrast to many other adaptor-cargo interactions (Traub and Bonifacino, 2013). Instead the N-terminal half of Synaptobrevin2’s SNARE domain itself mediates the adaptor binding. NMR (Koo et al., 2011) and crystallographic analyses (Miller et al., 2011) revealed a helical segment centered around M46 of Synaptobrevin2’s SNARE domain to constitute the binding surface, while on the adaptor site it is the ANTH domain which binds (Figure 2Ca). These data are in good agreement with earlier studies which had pinpointed M46 and D44 as determinants for Synaptobrevin2 targeting to synaptic-like microvesicles in neuroendocrine PC12 cells (Grote et al., 1995; Grote and Kelly, 1996). The interaction between the SNARE and the ANTH domain precludes complex formation between Synaptobrevin2 and t-SNAREs since the respective binding sites overlap. Thus AP180 and CALM can only facilitate the endocytosis of Synaptobrevin2 once the post-exocytic cis-SNARE complex has been disassembled by N-ethylmaleimide sensitive factor (NSF). In the context of selective Synaptobrevin2 sorting this is actually advantageous since only the v-SNARE Synaptobrevin2 should be targeted to new SVs while the t-SNAREs Syntaxin1 and SNAP25 should stay behind on the plasma membrane. At the same time AP180/CALM might act as chaperones which prevent the entry of Synaptobrevin2 into non-productive SNARE complexes while being sorted.

Another protein to which similar functions were ascribed is Synaptophysin1 (Rajappa et al., 2016). This second most abundant protein on SVs with still unclear function also interacts with Synaptobrevin2 (Calakos and Scheller, 1994; Edelmann et al., 1995; Washbourne et al., 1995). Like AP180 and CALM Synaptophysin1 can only bind to free Synaptobrevin2 (Edelmann et al., 1995; Siddiqui et al., 2007; Figure 2Cb). Therefore, it was also suggested to keep Synaptobrevin2 from untimely SNARE complex formation. More importantly, loss of Synaptophysin1 has been shown to slow the speed of endocytosis and more or less completely abrogate the internalization of Synaptobrevin2 in cultured hippocampal neurons (Gordon et al., 2011) based on Synaptobrevin2-pHluorin assays. In addition, the same group found maintaining the right stoichiometry between Synaptophysin1 and Synaptobrevin2 at a ratio of 1:2 to be critical for correct Synaptobrevin2 sorting (Gordon et al., 2016). Moreover, they showed mutations in Synaptophysin1 that are implicated in X-linked intellectual disability to impair Synaptobrevin2 retrieval (Gordon and Cousin, 2013).

The published effect of Synaptophysin1 loss on Synaptobrevin2 sorting is much more pronounced than the effect of the combined depletion of AP180 and CALM. This result seems puzzling in light of the fact that the AP180 KO mice have a postnatally lethal phenotype (Koo et al., 2015), while Synaptophysin1 KO mice display normal synaptic transmission (McMahon et al., 1996) and are indistinguishable from their littermates (Eshkind and Leube, 1995) except for their more exploratory behavior and some deficits in learning and memory (Schmitt et al., 2009). In line with this notion, two later studies reported only delayed endocytosis kinetics of several SV proteins (Kwon and Chapman, 2011) respectively only slightly less efficient retrieval of Synaptobrevin2 (Rajappa et al., 2016) upon loss of Synaptophysin1. To solve this discrepancy and dissect the contribution of Synaptophysin1 at the organismic level it will be important to generate mice with a combined deficiency in AP180 and Synaptophysin1 and to analyze whether they display an aggravated impairment of neurotransmission as compared to the AP180 KO mice. The same applies to the question of the relative contributions of CALM and AP180 to Synaptobrevin2 sorting.

## Sorting of Synaptotagmin1

Synaptotagmin1 is the major Ca^2+^ sensor on SVs and crucial for synchronous neurotransmitter release. Consequently, its absence leads to early postnatal death in mice (Geppert et al., 1994; Fernandez-Chacon et al., 2001). However, how Synaptotagmin1 couples Ca^2+^ influx to SNARE-mediated SV fusion at the molecular level is still not fully understood (Koh and Bellen, 2003; Koch and Holt, 2012), although a number of details have been unraveled. Synaptotagmin1 has a short, glycosylated luminal sequence followed by a single transmembrane domain and two characteristic cytosolic Ca^2+^ binding C2 domains (C2A and C2B). The binding of Ca^2+^ to both C2 domains affects the interaction of Synaptotagmin1 with SNARE complexes, even though it is still debated how exactly Ca^2+^ binding regulates full SNARE complex assembly (Jahn and Fasshauer, 2012). Moreover, incorporation of Ca^2+^ ions enables the C2 domains to associate with the plasma membrane, especially via binding to the phosphoinositide PI(4,5)P_2_ (Brose et al., 1992; Bai et al., 2002; Rhee et al., 2005; Park et al., 2015). Shielding of negative charges by Ca^2+^ binding even allows hydrophobic residues within Synaptotagmin1 to penetrate the plasma membrane (Bai et al., 2002). The insertion of Synaptotagmin1 into the lipid bilayer is believed to destabilize the membrane and thereby promote fast SV fusion and neurotransmitter release (Martens et al., 2007; Hui et al., 2009).

Loss of Synaptotagmin1 does not only result in reduced synchronous neurotransmission and increased asynchronous release, but also impairs compensatory endocytosis. In various model organisms the mutation, inactivation or deletion of Synaptotagmin1 caused endocytic defects (Jorgensen et al., 1995; Poskanzer et al., 2003; Nicholson-Tomishima and Ryan, 2004) possibly involving Synaptotagmin1’s Ca^2+^ sensing function since Ca^2+^ binding deficient Synaptotagmin1 mutants were unable to rescue the slow endocytosis kinetics (Yao et al., 2011). Given its essential function in exocytosis as well as endocytosis, Synaptotagmin1 has to be recycled with high fidelity during neurotransmission.

Synaptotagmin1 lacks the common endocytosis motifs for AP-2 interaction (Haucke et al., 2000; Grass et al., 2004), but can bind AP-2 via a basic stretch within the C2B domain. However, this interaction is not sufficient for mediating the endocytosis of the Ca^2+^ sensor, since Synaptotagmin1 when ectopically expressed in fibroblasts is not internalized from the plasma membrane (Feany et al., 1993; Jarousse and Kelly, 2001). Therefore, Synaptotagmin1 retrieval requires further apparently neuron-specific adaptors to accomplish efficient sorting.

The first functional link between Synaptotagmin and a specific endocytic adaptor was found in *D. melanogaster*. Genetic inactivation of StonedB resulted in severely paralyzed larvae due to defective neurotransmission (Grigliatti et al., 1973; Andrews et al., 1996; Estes et al., 2003) as a consequence of impaired SV recycling (Fergestad et al., 1999; Fergestad and Broadie, 2001). This led to the name “Stoned” for the affected locus (Grigliatti et al., 1973; Andrews et al., 1996; Estes et al., 2003). The synapses of the mutant larvae displayed in addition to a depletion of SVs a profound mislocalization of Synaptotagmin1 which was suggested to cause the also observed lower levels of the protein (Fergestad et al., 1999; Fergestad and Broadie, 2001). Overexpressing Synaptotagmin restored the embryonic lethality of StonedB mutants (Fergestad and Broadie, 2001) suggesting that sorting of Synaptotagmin is the major function of StonedB. Extensive biochemical analysis confirmed a direct interaction of StonedB as well as of its human homolog Stonin2 with Synaptotagmin in co-immunoprecipitation and GST-pulldown experiments (Phillips et al., 2000; Martina et al., 2001; Walther et al., 2001; Diril et al., 2006). Furthermore, transfected Stonin2 is recruited to the plasma membrane by ectopically overexpressed Synaptotagmin1 and allows its internalization in cell lines (Diril et al., 2006). Its enrichment in synapses further supported the idea that Stonin2 might serve as a specific cargo adaptor for Synaptotagmin1 (Walther et al., 2001; Diril et al., 2006). In fact, endogeneous Stonin2 also facilitated the endocytosis of Synaptotagmin1 in neuronal cultures (Kononenko et al., 2013).

Stonin2 and its homologs make up the Stonin protein family which is evolutionarily conserved from *C. elegans* to humans. While *D. melanogaster* and *C. elegans* possess with StonedB respectively Unc41 only a single stonin gene, mammalian genomes encode in addition to Stonin2 also the protein Stonin1. In their overall structure Stonin2 and its homologs resemble other endocytic adaptors like AP180 and CALM in having a folded cargo-binding domain and a presumably unstructured N-terminus for interacting with endocytic proteins (for a scheme see, Maritzen et al., 2010). WxxF motifs within this N-terminus allow Stonin2 to associate with the sandwich domain of the AP-2 α-appendage (Walther et al., 2004), while NPF motifs mediate its interaction with EF domains which are present in endocytic proteins such as Eps15 and Intersectin (Martina et al., 2001). The N-terminus is followed by a conserved Stonin homology domain (SHD) which is unique among the Stonin protein family (Maritzen et al., 2010), but does not yet have any assigned function. The C-terminus is structurally similar to the μ2 subunit of AP-2 and was therefore called μ-homology domain (μHD). It contains a short conserved tyrosine-based sequence motif that interacts with a basic stretch within the C2A domain of Synaptotagmin1 (Jung et al., 2007). This domain structure with its binding motifs enables Stonin2 to link Synaptotagmin1 to AP-2 and other components of the endocytic machinery and to thereby facilitate Synaptotagmin1 internalization (Figure 2Ba).

To fulfill its function Stonin2 needs to be localized to the periactive zone. The scaffold protein GIT (G protein coupled receptor kinase 2 interacting protein) which is associated with the cytomatrix of the presynaptic active zone (Kim et al., 2003) was shown to be crucial for recruiting Stonin2 there. In fact, GIT couples release sites to SV retrieval via its interaction with Stonin2. Mutations of the fly homolog dGIT in *D. melanogaster* severely reduce StonedB levels illustrating Stonin2’s dependence on GIT not only for its correct localization, but also for its stabilitzation. In line with the important role of GIT for Stonin2 function, dGIT mutants exhibit decreased Synaptotagmin1 levels thus mimicking phenotypes of the earlier described StonedB mutants (Podufall et al., 2014).

As loss of StonedB and Unc41 resulted in such severe phenotypes in invertebrates, their mammalian homologs were also expected to be essential for SV endocytosis (Stimson et al., 2001; Mohrmann et al., 2008) and Synaptotagmin1 sorting (Fergestad et al., 1999; Fergestad and Broadie, 2001; Mullen et al., 2012). In line with this, siRNA mediated knockdown of Stonin2 in cultured neurons was reported to slow endocytosis, initially suggesting also a major role for Stonin proteins in endocytic retrieval at mammalian synapses (Willox and Royle, 2012). However, quite surprisingly, a Stonin2 KO mouse model, which had been generated to dissect the role of Stonin2 at the organismic level, was viable and did not display any overt impairments (Kononenko et al., 2013) apart from an increased explorative behavior. Consistent with earlier results brain slices and cultured hippocampal neurons derived from Stonin2 KO mice displayed partial missorting of Synaptotagmin1, but not other SV proteins, to the surface of synaptic boutons underlining Stonin2’s role as specific adaptor for Synaptotagmin1 sorting during SV recycling. However, in striking contrast to reported phenotypes in invertebrates, SV endocytosis was rather accelerated than impaired, SV numbers were elevated, and the increased short-term facilitation and partial resistance to depression of Stonin2 mossy fiber synapses also pointed to an elevated SV pool size.

Compensation by another protein in mammals would be a likely explanation for the strikingly milder phenotype of Stonin2 loss in mice as compared to its lethal loss of function in invertebrates such as *D. melanogaster*. The closely related protein Stonin1 which according to its overall structure also appeared to be an endocytic adaptor was naturally the first candidate for compensating the loss of Stonin2. However, Stonin1/Stonin2 double KO mice did not display stronger brain phenotypes than the Stonin2 KO mice (Kononenko et al., 2013). Thus, Stonin1 obviously does not compensate for the loss of Stonin2 in Stonin2 KO mice, in line with the fact that it has not been detected in neurons so far. Indeed, in the meantime Stonin1 has rather been implicated in the sorting of the oncogenic proteoglycan NG2 in fibroblasts and in the regulation of focal adhesion dynamics (Feutlinske et al., 2015).

Interestingly, a peptide from the SV protein SV2 containing a tyrosine-based motif was shown to facilitate the internalization of Synaptotagmin1 by AP-2 (Haucke and De Camilli, 1999). These first biochemical data created the idea that in addition to the endocytic adaptor Stonin2 an SV protein, namely SV2, might play a role in Synaptotagmin1 sorting. SV2 comes in three isoforms termed SV2A, SV2B and SV2C that all share a 12-transmembrane domain structure. The SV2 protein family is conserved in vertebrates, but not in invertebrates. There is just one very distantly related protein present in invertebrates called SVOP (SVtwO related Protein). It displays only 20%–22% homology to SV2 and has a so far unknown function (Janz et al., 1998; Yao et al., 2013). Among the three vertebrate homologs SV2A is the only isoform with ubiquitous expression in the brain being present in glutamatergic as well as in GABAergic neurons (Buckley and Kelly, 1985; Bajjalieh et al., 1994). Although its function is still not fully understood (see Bartholome et al., 2017 for a recent review), SV2A plays a pivotal role in the CNS as its deletion in mice causes impaired neurotransmission and severe epileptic seizures culminating in premature death within 2–3 weeks after birth. This phenotype is not exacerbated upon loss of SV2B (Crowder et al., 1999; Janz et al., 1999) which is only expressed in glutamatergic neurons and absent from brain areas like dentate gyrus, globus pallidus and parts of the thalamus and substantia nigra (Bajjalieh et al., 1994).

All SV2 isoforms have been shown to interact with Synaptotagmin1 (Schivell et al., 1996; Lazzell et al., 2004; Schivell et al., 2005). Especially the N-terminus of SV2A was shown to bind to the C2B domain of Synaptotagmin1 (Figure 2Bb). This interaction is regulated by Ca^2+^ (Schivell et al., 1996) and is potentiated by phosphorylation of SV2A (Pyle et al., 2000). Among other residues threonine 84 of SV2A is phosphorylated by casein kinase 1 family kinases, and this phosphorylation is essential for Synaptotagmin1 binding and retrieval (Zhang et al., 2015), confirming a close functional relation between SV2 and Synaptotagmin1. Studying SV2A/B double KO mice Yao and colleagues found further evidence for a function of SV2 in Synaptotagmin1 sorting. Deletion of SV2A/B resulted in reduced Synaptotagmin1 levels, and the remaining Synaptotagmin1 was mislocalized to the surface of the presynapse at the expense of lower Synaptotagmin1 levels on SVs. Furthermore, they could show that the tyrosine-based motif within SV2A mentioned earlier is required for SV2A’s own endocytosis and for correct Synaptotagmin1 trafficking (Yao et al., 2010). All in all, SV2’s role in Synaptotagmin1 trafficking based on their biochemical interaction and *in vivo* data together with SV2’s appearance late in evolution, make SV2 a perfect candidate for compensating the loss of Stonin2 in Stonin2 KO mice. Similar to Synaptophysin1 presumably acting together with AP180/CALM in Synaptobrevin2 retrieval, SV2 and Stonin2 could have a partially redundant function in Synaptotagmin1 sorting in mammals (Figure 2Bc).

Indeed, consistent with this hypothesis the additional depletion of SV2A in Stonin2 KO mice exacerbated the phenotypes of the single mutants arguing for additive effects. The surface accumulation of Synaptotagmin1, the reduced Synaptotagmin1 levels and the impaired neurotransmission observed in SV2A/B double KOs were all aggravated in animals lacking SV2A/B and Stonin2. Furthermore, Stonin2 levels were elevated in SV2A/B double KO mice supporting the idea of a compensatory role for Stonin2 in the absence of SV2. In summary, SV2 and Stonin2 act in parallel to retrieve Synaptotagmin1, a mechanism specifically evolved in mammals to ensure precise sorting of this essential SV protein (Kaempf et al., 2015). The very distinct molecular features of both proteins enable them to bind to different C2 domains of Synaptotagmin1 eliminating competition for Synaptotagmin1 binding during its retrieval, which is consistent with their parallel mode of action (Figure 2B).

Interestingly, while Synaptotagmin1 is dramatically missorted upon loss of Stonin2 and SV2A/B, the kinetics of its retrieval are not slowed down (Kaempf et al., 2015). In contrast, using pHluorin reporters the endocytic retrieval kinetics of Synaptotagmin1 were shown to be even accelerated. This indicates for one that the speed of endocytosis and the efficiency of sorting can be uncoupled and second that increased levels of surface-stranded Synaptotagmin1 and faster retrieval kinetics correlate. Consistent with this notion overexpression of an internalization-defective mutant of Synaptotagmin1 in fact accelerated SV protein retrieval (Kononenko et al., 2013). Thus, surface-accumulated Synaptotagmin1 seems to serve as a trigger to initiate a faster mode of endocytosis. In the absence of this “signal” exocytosed SVs would predominantly be recycled via a slower endocytic mode. This is in line with the slow endocytosis kinetics observed in Synaptotagmin1 mutants or knockouts in *C. elegans* and vertebrates (Jorgensen et al., 1995; Nicholson-Tomishima and Ryan, 2004), but certainly needs further investigation to unravel how surface-localized Synaptotagmin1 can serve as a signal for regulating endocytosis kinetics. Clearly, future experiments are also needed to fully dissect the coordination between the parallel sorting of Synaptotagmin1 by SV2 and Stonin2. All in all, these results highlight the intricate interplay between endocytic adaptors like Stonin2 and SV proteins like SV2 to achieve high-fidelity sorting of crucial SV components such as Synaptotagmin1.

## Additional Functions for CME-Associated Adaptors: Release Site Clearance

Over the past 10 years evidence has accumulated that endocytic adaptors do not only function in the endocytic retrieval of SV proteins, but also in their fast clearance from release sites. Release site clearance might in fact constitute a greater bottleneck for sustained high-frequency neurotransmission than the recycling of SVs (Neher, 2010). Since synapses contain only a limited number of specialized release sites, these have to be reused repeatedly during high frequency stimulation (Neher, 2010). For the ribbon synapse of inner hair cells which can release hundreds of SVs per second it is estimated for instance that the individual release sites have to be used up to 10 times per second (Jung et al., 2015). This means there is a maximum of about 200 ms available for returning release sites back to fusion-competence after SV exocytosis (Neher, 2010) i.e., for clearing away previously exocytosed proteins. This interval would be compatible with UFE, but not with slower modes of CIE. However, since UFE is described to occur at sites adjacent to the active zone (Watanabe et al., 2013), the question still remains how the exocytosed SV proteins rapidly leave the release site to make space for the next round of SV fusion.

The first implication for a role of endocytic proteins in this process came from studies of the temperature-sensitive *D. melanogaster* mutant “shibire” (Kawasaki et al., 2000) which impairs Dynamin function. When challenged at the non-permissive temperature with a 50 Hz stimulus train, these flies exhibited already at the second response after only 20 ms a reduction in their synaptic current amplitude. This short term synaptic depression was much too rapid to be explained by a depletion of the SV pool and argued for an additional role of Dynamin prior to SV recycling (Kawasaki et al., 2000). Studies at different synapses have hence confirmed that interfering with different endocytic proteins has a rapid effect on exocytosis eliciting short term depression independent of the SV resupply (Shupliakov et al., 1997; Chen et al., 2003; Ferguson et al., 2007; Hua et al., 2013). Using the calyx of Held as their model system Hosoi et al. (2009) showed that not only impairing Dynamin function delays the recruitment of release-ready vesicles, but also disrupting the interactions between Synaptotagmin1 and endocytic factors or between different endocytic proteins. Deletion of the endocytic scaffold Intersectin1 was likewise shown to cause short term depression at the calyx of Held without significantly influencing the rate of endocytic membrane retrieval (Sakaba et al., 2013). Finally, inner hair cell synapses from conditional AP-2μ KO mice exhibited slowed release immediately after fusion of the readily releasable pool of vesicles which is consistent with a delayed reuse of release sites (Jung et al., 2015). However, since the level of the AP-2 cargo otoferlin, which is implicated in SV priming, is strongly reduced in AP-2 deficient inner hair cell synapses, a priming defect could also explain the early onset release impairment. It is a general problem that release site clearance defects can rarely be unambiguously distinguished from problems in SV priming with the available techniques.

How might endocytic proteins help to clear away newly exocytosed SV proteins that are clogging release sites? A possible mechanism is lateral sequestration where endocytic proteins indirectly provide directionality to the random diffusion of newly exocytosed SV proteins by capturing and concentrating them at the edge of the active zone. In line with this, Monte Carlo simulations by the Klingauf group show that a trapping “sink” prevents freely diffusing particles from a random walk back to the source and thereby speeds up their clearance (Rajappa et al., 2016). Supporting evidence for such a model comes not only from their own data (Rajappa et al., 2016), but also from a recent study dissecting the fate of newly exocytosed phluorin-tagged SV proteins (Gimber et al., 2015). Immediately post-fusion Synaptobrevin2-pHluorin molecules were shown to freely diffuse and thereby rapidly disperse. This initial short phase of free diffusion was followed by confinement and slow reclustering at the periactive zone. For Synaptobrevin2 this confinement was shown to depend on its interactions with its endocytic adaptors AP180 and CALM, presumably aided by a presynaptic diffusion barrier of unknown identity (Gimber et al., 2015).

Based on this example the CME machinery is anticipated to promote release site clearance by sequestering SV proteins. Analogous to AP180 and CALM helping to confine their cargo Synaptobrevin2 (Gimber et al., 2015), AP-2 is proposed to facilitate lateral sequestration of its cargo Otoferlin at inner hair cell synapses (Jung et al., 2015), however this hypothesis has not been tested so far. For proteins like Intersectin1 and Dynamin which do not have an explicit cargo sorting role in CME, it is less clear how they might facilitate the translocation of SV proteins away from the active zone. For Dynamin it was speculated that the mechanoenzyme might fulfill a potential requirement for membrane remodeling in release site clearance (Sakaba et al., 2013). Since Intersectin1’s function in release site clearance involved its binding to the small GTPase CDC42, an Actin regulator, it was hypothesized that Intersectin1 might regulate Actin-driven movement of an endocytic complex out of the active zone (Sakaba et al., 2013). Being a large endocytic scaffold with numerous protein interaction surfaces Intersectin1 might in fact associate with a so far unidentified SV component and facilitate its specific clearance. In addition, the non-cargo adaptors that are involved in release site clearance might contribute to the network of low affinity interactions that keep the different CME components recruited to the periactive zone and thereby stabilize the presence of the endocytic adaptors there. In addition to promoting release site clearance the sequestration of SV proteins by adaptor proteins counteracts their unrestricted diffusional escape into the axon which would prevent their efficient retrieval for SV recycling.

Not only endocytic adaptor proteins are important for release site clearance, but SV proteins themselves help to prevent short term depression. Rajappa et al. (2016) recently discovered that dimerization of Synaptobrevin2 and its association with Synaptophysin1 are crucial for efficient removal of newly exocytosed Synaptobrevin2 from the active zone. Synaptobrevin2 and Synaptophysin1 reside in SV protein clusters outside the active zone termed the “readily retrievably pool” (RRetP) for subsequent endocytosis (Wienisch and Klingauf, 2006). There Synaptophysin1 acts as an additional sink for binding newly exocytosed Synaptobrevin2. Accelerating Synaptobrevin2’s removal is especially important to prevent it from clogging the release site by entering into cis-SNARE complexes with the abundant t-SNAREs (Rajappa et al., 2016).

However, what is happening to the cis-SNARE complexes that are already present? How are they kept from jamming the release site? The association of Synaptobrevin2 with its adaptors AP180 and CALM or with Synaptophysin1 cannot facilitate the removal of cis-SNARE complexes since these interactions only work with non-SNARE-complexed Synaptobrevin2. Principally, cis-SNARE complexes are disassembled by the ATPase NSF post-fusion (Jahn and Scheller, 2006; Zhao et al., 2015), but the disassembly process was estimated to take, at least *in vitro*, about 5 s (Cipriano et al., 2013) i.e., much more time than available for release site clearance during high-frequency stimulation. Therefore, it is surprising that no mechanism, no “sink”, has been discovered so far to accelerate the translocation of intact cis-SNARE complexes to the periactive zone where there should be more time available for their disassembly. Interestingly, Synaptobrevin2 presumably contains with its extreme N-terminus a site that should still be accessible for interactions after SNARE complex assembly. Infusing this proline-rich domain into the calyx of Held impaired the replenishment of fast releasing vesicles (Wadel et al., 2007). Wadel et al. (2007) concluded that the “proline-rich domain of Synaptobrevin2 is important for recruiting rapidly releasing SVs”. This could for instance be the case if it facilitates the clearance of cis-SNARE complexes from release sites, a question which deserves further investigation.

## Summary and Outlook

Inspite of decades of research the question how SVs are recycled is still not fully resolved. The last years have rather expanded the number of suggested recycling modes instead of pinpointing the one single mechanism that is operating under all conditions. This is most likely due to the fact that there is no single mechanism. In fact, most studies agree first of all in their observation of more than one time constant of SV retrieval (Sun et al., 2002; Gandhi and Stevens, 2003; Wu et al., 2005; Delvendahl et al., 2016; Soykan et al., 2017). While (ultra-)fast retrieval appears to operate in response to stimulation with one or few action potentials, endocytosis is reported to slow down with increasing stimulation strength and frequency ((Sun et al., 2002; Delvendahl et al., 2016; Soykan et al., 2017), but see also (Wu et al., 2005)). Thus, SV recycling like so many other neuronal pathways is a plastic process that can be fine-tuned to the need of the respective neuron in a specific situation. Key questions now are: Why is there a limit to the capacity of the fast retrieval mode? Are there preformed structures that are used for rapid retrieval? How is exocytosis coupled to the different retrieval modes? How is the switch between modes regulated by neuronal activity? Do Ca^2+^, phosphoinositides, SV protein surface levels or membrane tension play a decisive role in this regulation? Do different types of synapses prefer different retrieval modes? In how far do fast and slow retrieval use the same molecular machinery? How is adaptor-mediated SV protein sorting integrated into the different retrieval modes and at which location does the bulk of SV protein sorting take place? Do cargo proteins differ in respect to when and where they are sorted? For addressing these questions highly time- and space-resolved microscopical techniques which can monitor membranes and proteins simultaneously and live will be needed to overcome the limitations of earlier approaches which are either blind to proteins (e.g., capacitance measurements) or to membranes (e.g., pHluorin probes). Besides a systematic study of different synapse types using a range of stimulation paradigms together with genetic manipulation will be crucial to unravel synapse-specific and activity-dependent adaptations of SV recycling.

Even though our concepts of SV retrieval have undergone a substantial revision over the past years, the importance of endocytic adaptor proteins for safeguarding sustained neurotransmission remains undisputed. They are rather now appreciated to fulfill a number of additional functions besides linking exocytosed SV proteins to the CME machinery for efficient internalization. For one, we now know that the adaptors, including AP-2, together with Clathrin and other endocytic factors not only act at the plasma membrane, but also on ELVs. This raises a plethora of questions starting with the lipid identity of this compartment. Super-resolution microscopy or correlative light electron microscopy in conjunction with suitable phosphoinositide probes will be needed to address this question. It will also be important to unravel how ELVs communicate with other endosomal compartments that have been implicated in SV recycling, especially as the question of SV protein quality control is largely unresolved. Are there specific mechanisms to ensure that damaged SV proteins or SVs that do not have the required composition are disposed off? Second, adaptor proteins are involved in release site clearance, even though the exact mechanism has not yet been proven. However, it seems likely that they facilitate the capture of newly exocytosed freely diffusing SV proteins at the periactive zone thereby accelerating their removal from release sites. To address this point in detail more super-resolution microscopy studies are needed which follow the fate of freshly exocytosed SV proteins and endocytic factors with high spatiotemporal resolution. Finally, in SV protein sorting for endocytosis as well as in release site clearance adaptor proteins were shown to work closely together with the SV proteins themselves. Theoretical considerations suggest that high fidelity SV protein retrieval cannot be accomplished solely via adaptor-based sorting (Gauthier-Kemper et al., 2015). Therefore, the combined action of SV protein self-assembly mechanisms, as exemplified by Synaptobrevin2 and Synaptophysin1 oligomerization, and of specific SV protein recognition by adaptors, as illustrated by AP180-mediated Synaptobrevin2 sorting, is likely key to the successful clearance and retrieval of the full set of SV proteins.

## Author Contributions

NK and TM conceived and wrote this review article.

## Conflict of Interest Statement

The authors declare that the research was conducted in the absence of any commercial or financial relationships that could be construed as a potential conflict of interest.
